# Re-Design of Machine Tool Joint Components Based on Polymer Fillings for High-Speed Performance

**DOI:** 10.3390/ma14226913

**Published:** 2021-11-16

**Authors:** Zuzana Murčinková, Pavel Adamčík, Jozef Živčák

**Affiliations:** 1 Department of Design and Monitoring of Technical Systems, Faculty of Manufacturing Technologies with Seat in Prešov, Technical University of Košice, Bayerova 1, 08001 Prešov, Slovakia; 2 Technická Diagnostika, Ltd., Jilemnického 3, 08001 Prešov, Slovakia; p.adamcik@diagnostika.sk; 3 Department of Biomedical Engineering and Measurement, Faculty of Mechanical Engineering, Technical University of Košice, Letná 9, 04200 Košice, Slovakia; jozef.zivcak@tuke.sk

**Keywords:** polymer filling, polymer concrete, testing station, amplitude of vibration, acoustic emission, high frequency, damping properties, reduction in dynamic response

## Abstract

In this paper, we report the results of an experimental study of a re-design approach using filling polymers and particulate composites with a polymer matrix, thus creating a macroscopic hybrid structure. The re-design is focused on the joint of a textile machine. It is a re-design of already existing machine parts of a joint in order to increase the damping of components, reduce the amplitudes of high-frequency vibrations and acoustic emission for high-speed operation of textile rotors, and to compare individual structural modifications of the rotor housing body and absorber of high-speed textile rotor in a spinning unit with respect to dynamic properties of that measured mechanical system. The experiments included a bump test, determination of logarithmic decrement, measurement of vibration acceleration, a wavelet analysis, and measurement of acoustic emission. When excited by high frequency signal amplitudes up to 5 g, the benefits of polymer filling were manifested by an approximately 50% reduction in amplitude vibrations, a 66% reduction in acoustic emission amplitude, and an 85% reduction of the maximum peak in the acoustic emission FFT spectrum. In the area above 10 g, the stiffness of the component dominated to reduce the magnitude of vibrations.

## 1. Introduction

The achievement of high-performance production requires an increase in the operating speed of machines. At the same time, by increasing operating speed, unacceptable machine vibrations can be created, due to unfavourable running conditions in the case of both classical and modern production machines. Thus, as production speeds increase, there is a need for the increased damping of unwanted vibrations.

One of the possibilities is the use of material damping. However, materials with excellent material damping have low stiffness. Therefore, the concept of combining different types of materials in the form of hybrid structures has emerged. Regarding the variety of available materials, essentially, metal, stone, ceramic, polymer concrete, porous, and reinforced composite materials can be seen in machine tools and components [1].

Modern production machines are those with lightweight components that are used to increase the energy efficiency of machines. Usually, the realization of lightweight design in machine tools can be achieved using strategies of structural, material, and system lightweight design [2]. However, the trend of using lightweight components encounters the physical limits of standard existing materials, which have either high weight and at the same time rigidity, or vice versa. Depending on the specific application, material engineers develop materials with conflicting properties, namely high rigidity and low weight. Moreover, in addition to stiffness and density, damping is the third key factor that influences the dynamic stiffness of frame structure [3].

Conventional machine tool designs, based mainly on the use of conventional construction materials (steel, cast iron, and aluminium alloy), can be adapted to reduce vibration amplitudes, for example, due to increasing operating speeds. In the case of existing production machines, mainly for economic reasons, a simple approach is needed in the shortest possible time. Such an approach with respect to higher rigidity and/or weight to reduce unwanted vibrations does not involve developing new structural designs based on changing the shape of components, their internal topology, or their optimisation; however, it involves their design based on the addition of damping material applied in the cavities of the individual components, in order to reduce high frequency vibrations, which is the subject of this article.

Conventional cement concrete was used in machine building (not as a filler) as a substitute for cast iron after World War I and during World War II, due to a lack of metal material, but not due to increased damping [1,4]. The idea of filling the cavities of production machines began to be applied in the 1980s–1990s, when cast iron, and later lighter welded steel structures, were filled mainly with sand, oil, or foams, as well as with special concrete in order to increase structural damping. Thus, the level of static and dynamic rigidity of machine tool structure was improved.

The mechanism of vibration damping in polymers at the molecular level is described in [5] where the long-chain molecules of polymers are compared with the crankshaft mechanism, in which the molecules are assumed to be a series of jointed segments that have some degrees of freedom for movement. The long-chain molecules need more energy to vibrate than that required by small molecules [6].

The high damping property of polymer concrete is assumed to be caused by the viscoelastic nature of polymeric resin, filler particles, internal interfacial area, and internal defects, for example, air voids [5,7]. In the case of the propagation of waves caused by vibrations, the mentioned sources of damping, especially reinforcing particles or short fibres, contribute to the absorption of vibrational energy. Numerical simulations of damping ratios of stress waves for high velocity shock waves in fibre composites have shown an increase in damping up to 37% (fibre volume 35%), as comparing with a model without fibres [8,9].

Current research in the field of polymer concretes as particulate reinforced polymer composites has focused on evaluating mainly mechanical, dynamic, and thermal properties such as tensile strength, compressive strength, flexural strength, deflexion, the coefficient of thermal expansion, damping characteristics, hardness, failure modes, and impact energy absorption. Significant damping properties of polymer concrete are known, and published research results in this field are, for example, in [7,10,11,12,13].

Furthermore, developments in the field of polymer composites have focused on testing different volume fractions of individual components, filler sizes, and their optimum composition, such as in [10,14,15], and the use of components such as basalt, spodumene, fly ash, chalk, metakaolin, recycled glass sand [10,16,17], short natural fibres (jute fibres and ramie fibres) [18,19], short nettle fibres [20], basalt fibres [21], marble and quartz powder [11], glass powder [22], unusual shapes (aluminium polymer hybrid foam with the foam sphere filler [23], hollow spheres [24], and with matrices of different resins (polyester, vinyl ester, and epoxy) [16]. 

Research using polymer concrete for a machine-base frame or to fill existing machine designs for improvement of dynamic stiffness has been investigated in advanced applications of production machine components for precision tool machines [10,13], a grinding machine [5], and a machine tool worktable [21]. In [1], the designs of various machine components were reviewed based on the development of material characteristics, structure of materials (foams, composites, and hybrids), and structural topology of components with comparative studies. Composite metal hybrid structures have been fabricated from welded steel beds filled with composite or viscoelastic material. The hybrid structure resulted in an increase in the damping capacity, the natural frequency of the corresponding mode shapes of the machine tool body [25,26,27]. The performance of a grinding machine hybrid structure has been evaluated using surface roughness [5]. The use of a polymer concrete bed of the CNC turning centre has resulted in improved behaviour in terms of tool wear and surface roughness [28].

Naturally, the design of machine tools is supported by simulation approaches and structure optimization, described in [29,30,31], and focused mainly in stiffness. However, computations of composite materials and hybrid structure materials require advanced models and computation, i.e., multilevel models, representative volume element models, micromechanical models, etc., using finite element analysis, boundary element methods, or meshless methods focusing on linear, nonlinear static, dynamic analyses, and optimisation. Experimental studies have mainly been based on an experimental modal analysis, measurement of vibrations and analysis of dynamic signal, acoustic emission analysis, temperature measurements, etc., with the goal of finding correlation data between testing, and simulation results and obtaining the optimised structure, such as described in [32,33]. Many other studies or virtual testing of machine tools and their frames and modules with the aid of computational methods are described in [10,13,27,34], as well as with a stochastic approach in [35].

The contribution of this study is the application of a re-design approach using polymers and particulate composites with a polymer matrix as a cavity filling. The filling is applied to the individual components of a production machine joint, which is not a frequent application. Usually, this approach is applied to a base frame. Furthermore, polymer concrete is usually used as the structural material of individual machine components. In this study, polymers and polymer concrete are used as a component filling, creating a hybrid structure in a production machine joint. Thus, the volume of supplemented polymer is small as compared with the volume of other metal components. The focus of this study is to re-design existing machine parts using polymers to reduce the amplitudes of high frequency vibrations for high-speed operation. The aim is to compare the individual structural modifications of components of a textile machine spinning unit with respect to their dynamic properties.

## 2. Description of the Measurement, Materials, and Re-Designed Components

The requirement was to perform a re-design of selected components of a spinning unit joint so that the roller bearing (rotor) performance could operate with a magnitude of vibration below the prescribed Warning and Danger operation mode limit, i.e., a vibration acceleration amplitude less than 5 g and above 10 g, respectively, preferably at the highest possible rotational speed. 

**Figure 1:**
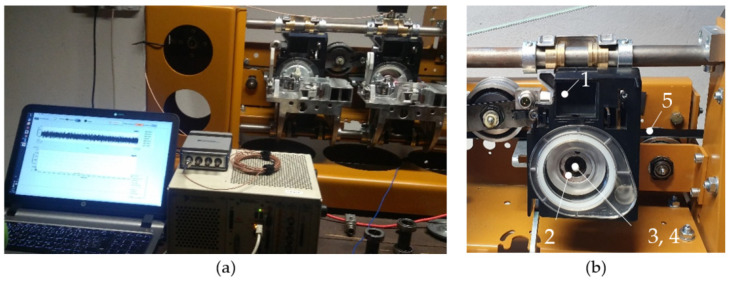
(**a**) Testing station and part of the measuring chain components; (**b**) the spinning unit 1, rotor housing body 2, rotor 3, absorber 4, and flat belt 5.

Measurements were performed on a testing station (Figure 1) using the following hardware and software:

− An accelerometer PCB model 352A60, PCB Piezotronics, Depew, NY, USA, with a frequency range up to 65 kHz and a sensitivity of 10 mV/g; Dynamic Signal Acquisition device PXI-4462 PXI Sound and Vibration Module Meter, National Instruments Corporation, Austin, TX, USA, A/D converter resolution 24-bits, sample rates, samples-per-second 1 kS/s to 204.8 kS/s in 181.9 μS/s increments;
− Acoustic emission sensor Vallen-VS45-H with the range of 20 kHz–400 kHz, Dynamic Signal Acquisition device NI-9223 module meter, signal level ±10 V, resolution 16 Bit, sample rates 1 MS/s/ch, National Instruments Corporation, Austin, TX, USA;
− Software for advanced analysis of the dynamic signal base on LabView Sound and Vibration Toolkit software, National Instruments Corporation, Austin, TX, USA.

**Figure 2:**
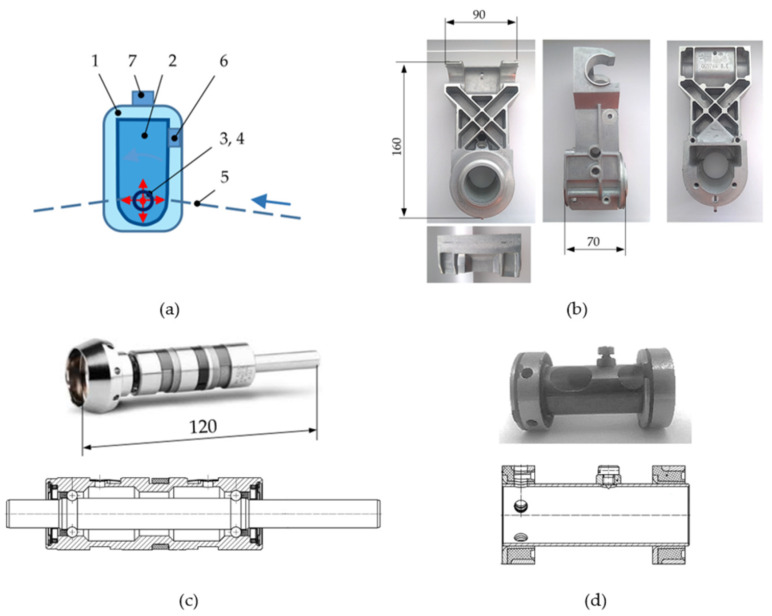
(**a**) Schema of the spinning unit 1, rotor housing body 2, rotor 3, absorber 4, flat belt 5, accelerometer 6, and acoustic emission sensor 7; (**b**) rotor housing body; (**c**) rotor with cup head and its cross-section; (**d**) absorber and its cross-section.

The spinning unit 1 in Figure 1b and Figure 2a consists of a rotor housing body 2 with a high-speed rotor 3 (Figure 2c) driven on its end-pin by a flat belt 5. The rotor 3 is housed in an absorber 4 (Figure 2d), which consists of a steel thin-walled tube and, at its ends, consists of steel bushings filled with rubber. The components 3 and 4 are both housed in a hole in the rotor housing body 2 (Figure 2b), which is mounted to the rod of the testing station (representing the rod of the textile machine line). The rotor housing body and absorber are static. 

Rotor 3 is not a standardized bearing. It acts as a rotor (spindle) and it is designed for textile machines with a flat belt drive. The rotor consists of an outer case and two separate rows of rolling elements (balls) in cages, and a shaft, one pin of which is adapted for a flat belt and another pin for pressing the cup head (Figure 2c). The outer diameter of the rotor is 22 mm and the shaft diameter is 8.5 mm. The radial clearance is set by the producer and its prescribed range is 4 to 8 μm. The precision grade of a high-speed rotor is 5–10 times higher than the standard production of bearings.

The mechanical system of the testing station and its components were excited in the rotational speed range of 70,000–135,000 min^−1^ by gradually increasing the rotational speed for 80 s. In this way, a response could be observed for different rotational frequencies in the range of 1167–2250 Hz. Mechanical vibrations and acoustic emission were measured in a radial direction using an accelerometer 6 and acoustic emission sensor 7 (Figure 2a). 

All measurements were made using the new rotors, without signs of damage (confirmed after disassembly), rotors were after inrun and the control measuring by producer, and the quality of raceways was in their original state, i.e., as they are after production. According to the operating state of the rotor, the measurements were made for two main modes, namely excitation:

Mode 1: Low amplitudes, i.e., up to 5 g, which corresponds to suitable rotor operating conditions and a satisfying safe operation mode;

Mode 2: High amplitudes, i.e., above 10 g, which corresponds to unsuitable rotor operating conditions and the mode Danger.

We focused on two extremes modes, i.e., satisfying and dangerous operation. The amplitudes between 5 g and 10 g correspond to the mode Warning and the corresponding excitation was not used and measured. 

The sources of excitation come from the high-speed operation of the flat belt and the rotor. The flat belt pressing force on rotating pin of rotor is 12 N ± 2 N. The change of the flat belt pressing force causes the change of contact angle between rolling ball and raceway and thus the kinematic slippage is changed. The slippage appears mainly at rotational speed 110,000–135,000 min^−1^. The dynamic unbalance is not the main source of excitation. The dynamic unbalance causes 3–5% portion of the measured amplitudes. The very precise dynamic balancing of the rotor is made before usage to meet the strict prescribed residual dynamic unbalance, i.e., up to 0.02 g mm (gram-millimetres). 

The mentioned effects of flat belt and rotor high-speed performance are involved in character of periodic and polyharmonic excitation force *F*(*t*). The well-known equation of motion for a driven and damped oscillator can be written in the form involving the quality factor *Q*, the coefficient of performance, according to [36] as

(1) $$\ddot{x}+\frac{\omega_{0}}{Q}\dot{x}+\omega_{0}^{2}x=\omega_{0}^{2}\frac{F(t)}{k},\;\mathrm{with}\;\omega_{0}^{2}=\frac{k}{m}$$

where $x,\dot{x},\ddot{x}$ are displacements, velocities and accelerations, respectively, *ω*_0_ is undamped natural frequency, *F*(*t*) is external force, *m* is mass, and *k* is spring constant. Quality factor *Q* is, in general, defined as 2π times the ratio of the energy of the oscillator to the energy lost to friction per cycle [36]. Depending on the value of *Q* (one of damping measures), the motion is either overdamped, critically damped, or underdamped.

**Figure 3:**
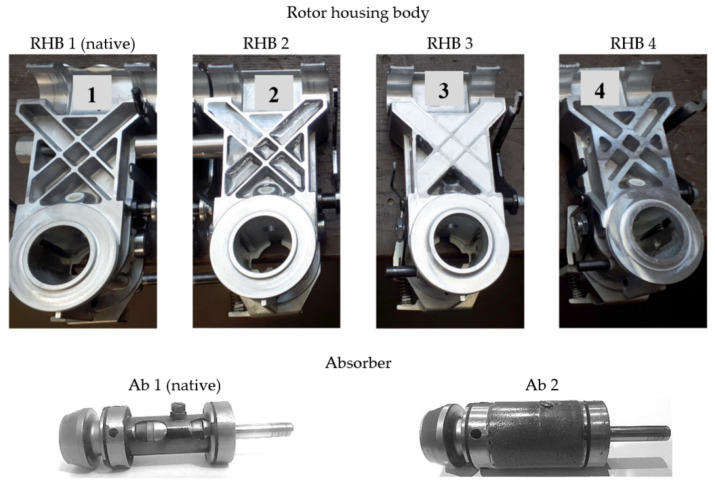
Rotor housing body (RHB) and absorber (Ab); native and the experimental re-designs.

The measurement was performed by comparing the native designs (RHB 1 and Ab 1 in Figure 3) and variants of the rotor housing body design (RHBs 2–4 in Figure 3) and absorber (Ab 2 in Figure 3) mounted in the spinning unit. The measurement of the re-designed absorber Ab 2 was for mounting in the native variant of the rotor housing body, i.e., RHB 1. We evaluated the reduction in vibration amplitudes by comparing behaviours without damping material and the effect of adding two types of polymeric damping materials A and B (epoxy and silane resins) differing in the filled volume in the rotor housing body, and damping material C (polymer concrete) in the absorber (Table 1).

The material of the native rotor housing body is an aluminium alloy (Table 1). For high-frequency vibrations, the material damping of the aluminium alloy and the rigidity of the structure are not sufficient. It is known that with increasing excitation frequency, the there is a decrease in the values of the damping parameters. By adding damping material A and B and C, a hybrid structure was created, i.e., the stiff base structure with low material damping was filled with a material with high material damping. Similarly, the free volume of the native absorber component was filled with damping material C, i.e., polymer concrete as particulate composite with polymer matrix, with the following composition: silica sand, matrix 2:1; sand fraction 0.4–0.8 mm; epoxy resin matrix LR285; and the curing agent LH285. Compared with native RHB 1, the mass of RHB 2 was 12% higher and the weights of RHBs 3 and 4 were 33% higher. The masses of RHBs 3 and 4 were the same. The mass of native absorber Ab 1 increased by 72%.

## 3. Re-Designed Rotor Housing Body and Results

The following section presents the results and analysis for the rotor housing body of the spinning unit with thin and thick ribs, with the filling of the damping materials A and B (according to Figure 3 and Table 1), with evaluation of the natural frequencies by the bump test and acceleration of vibrations during excitation in the range of 70,000–135,000 min^−1^ for the above-mentioned two excitation modes.

### 3.1. Bump Test

**Figure 4:**
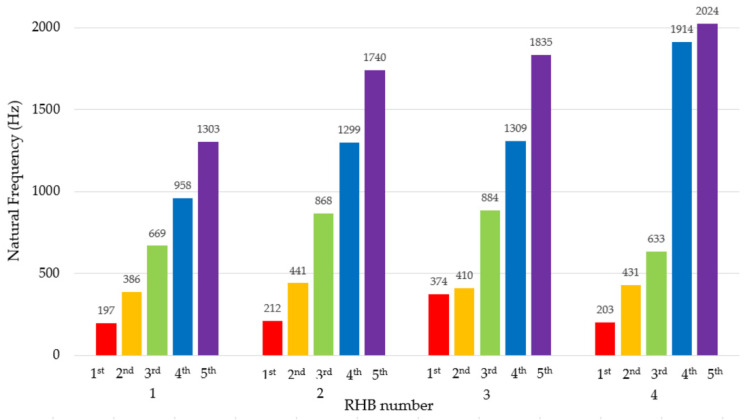
Natural frequencies (1st–5th) and vertical, horizontal, and axial directions of impacts in the bump test (up).

It is known that material is one of the parameters that changes the natural frequency. By using a bump test and evaluating the excited natural frequencies, it can be confirmed that the natural frequency is changed. Three impact tests were performed on each rotor housing body when hanging it free to bump in the vertical direction (to the upper flat surface of the body), in the horizontal direction (to the lateral flat surface of the body), and in the axial direction (to front of the cylindrical part of the body), (Figure 4, up). The modal hammer tip that was used to make the bump was made of hard plastic.

The most significant difference in the first natural frequency is for the hybrid structure formed by the damping mass B and the native aluminium alloy structure (RHB 3). The first natural frequency of the native RHB 1 is 197 Hz. The epoxy resin filling the front cavities (RHB 2) minimally changed the first natural frequency (212 Hz). In the case of RHB 3, the first natural frequency of this body is 90% higher (374 Hz). Modifying the native RHB 1 by increasing the thickness of the ribs (RHB 4) mainly changed the third and higher natural frequencies.

The logarithmic decrement values of the individual bodies in Table 2 were determined by the method of the time response of the free damped vibrations induced by the bump test impulse. Damping materials A and B, i.e., epoxy and silane resins, increased the damping measure and logarithmic decrement values by 15% and 62%, respectively. Increasing the logarithmic decrement also shortens the damping time. The volume of silicone damping material B is larger, as both the front and back cavities of the rotor housing body are filled. Although it is a larger added volume, considering the first natural frequency and the logarithmic decrement, the silane damping material appears to be more advantageous.

### 3.2. The Response to High-Frequency Excitation

**Figure 5:**
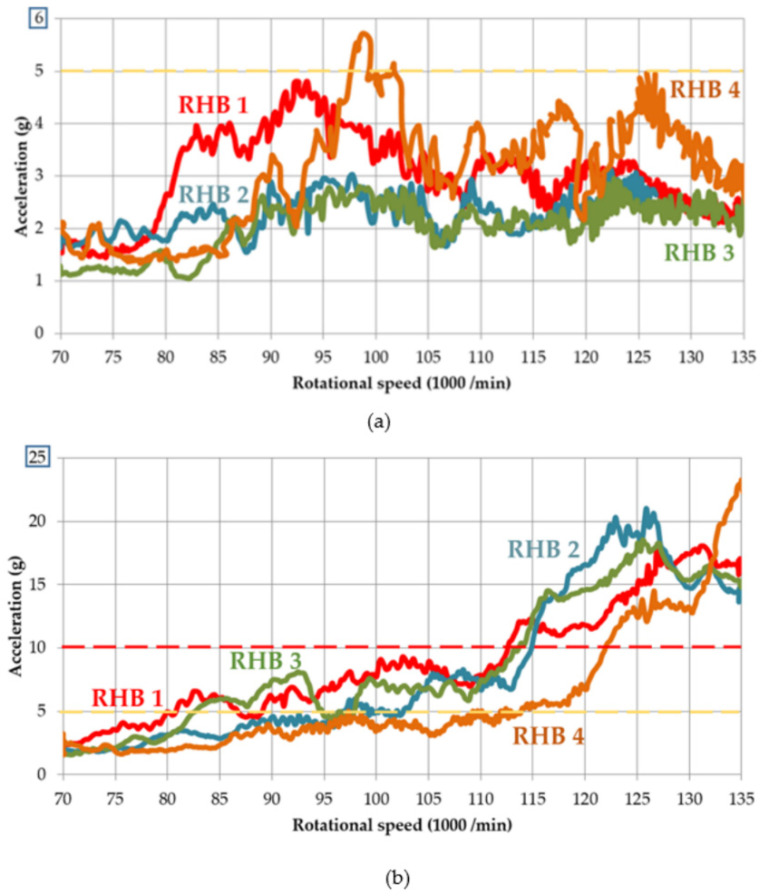
Vibration acceleration for RHBs 1–4: (**a**) excited up to 5 g; (**b**) excited above 10 g.

Figure 5 shows the comparisons of the vibration accelerations for the RHBs 1–4 with a rotor generating a dynamic signal with a Mode 1, i.e., low vibration amplitude, less than 5 g (Figure 5a) and with a Mode 2, i.e., rotor generating a high vibration amplitude greater than 10 g (Figure 5b) for excitation in the rotational frequency range 1167–2250 Hz. The measurements were made in triplicate for each group. When excited by different pieces of rotor, the response characteristics were similar to that shown in Figure 5.

In the area of safe operation (under the yellow dashed line, Figure 5a), RHBs 2 and 3 with damping materials A and B, respectively, have lower values of vibration acceleration and summation value in the entire range of the excitation rotational frequencies 1167–2250 Hz. At the same time, the stabilisation of the response, i.e., the difference between the maximum and minimum values at the mentioned excitation rotational frequencies is significantly smaller for RHBs 2 and 3. There is a significant reduction in the dynamic response using the damping materials, which are approximately 50% of the mean value of the vibration acceleration, as compared with the rotor housing body without damping mass. RHB 4, with the thick ribs and highest stiffness, has a comparable response in the area of safe operation; as the native design, there is no clear benefit.

However, when excited by a rotor with a high amplitude vibration (Figure 5b), a significantly better design cannot be evaluated among RHBs 1, 2, and 3. RHB 4 with thick ribs provides the lowest vibration acceleration values. Up to a speed of 113,000 min^−1^, the values are below the warning limit even for excitation by a rotor of amplitudes above 10 g. RHBs 1–3 reach this limit at 80,000–98,000 min^−1^.

Although the weight of RHB 3 with the damping material B and RHB 4 with the thick ribs is the same, the effect of increased body stiffness is more suitable when excited by high amplitudes, and the presence of a damping material is suitably manifested at low amplitudes of high frequency vibrations. This corresponds to the measured logarithmic decrement values in Table 2.

### 3.3. Wavelet Transform

**Figure 6:**
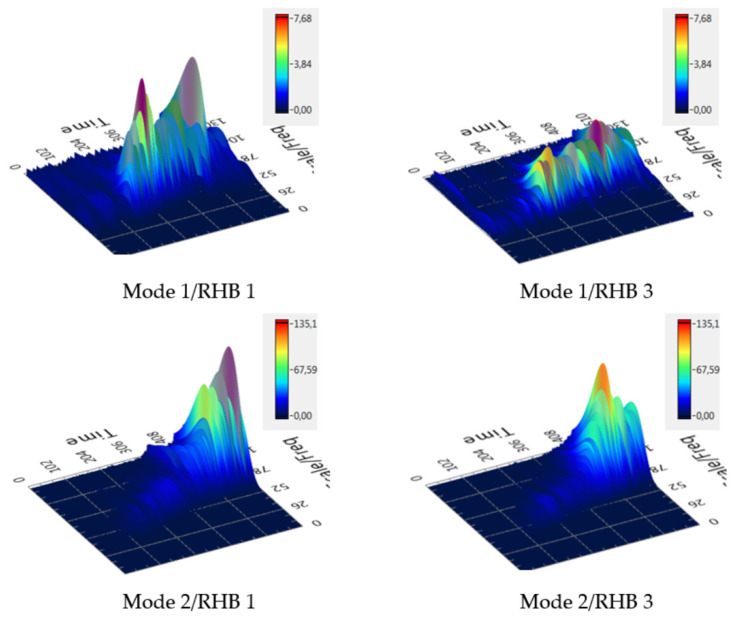
Wavelet transform in 3D view, frequency (Hz), time (s), and amplitude (g).

The vibrodiagnostic signal of complex systems with rotating parts involves a large amount of information and there are several ways to evaluate it. A comparison of the dynamic response of RHBs 1 and 3 is also shown using the processing of the vibrodiagnostic signal using the wavelet transform (Figure 6). This is a wavelet transform [37], which evaluates the signal over the time and frequency domains; thus, changes over time can be observed, which is suitable for non-stationary signals, such as in this study. In this way, the transient information can be detected in the measured signal. Figure 6 shows the comparison of the native design (RHB 1) and with the filling with the damping material B (RHB 3).

By comparing the wavelet transform graphs for low and high vibration amplitude excitation of RHBs 1 and 3, i.e., without and with the damping mass, a much “calmer” response (lower amplitudes) can be observed in the case of RHB 3 with damping material B. 

**Figure 7:**
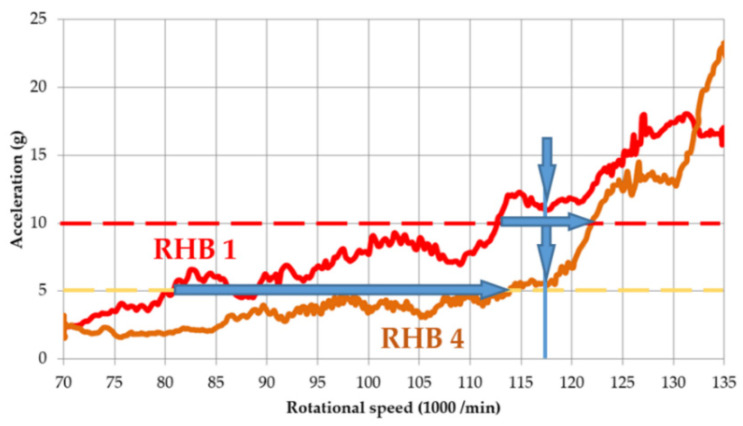
Comparison of vibration acceleration for RHBs 1 and 4 for excitation above 10 g.

Naturally, when comparing the designs of RHBs 1 and 4, i.e., the thin-ribbed body and the thick-ribbed body, in terms of vibration amplitudes, the thick-ribbed RHB 4 behaves more suitably due to the higher rigidity of the body as well as the weight. Its first natural frequency is only 3% higher, and its weight is 33% higher. Vibration measurements show that the vibrations of the thick-ribbed RHB 4 are lower over the whole range of excitation rotational frequencies. The biggest difference is at the rotor speed of 117,500 min^−1^, when RHB 4 has a vibration acceleration that is 50% less than RHB 1 (Figure 7, indicated by arrows). For a body with thick ribs, the limits of safe operation and of danger are shifted by 40% and 10%, respectively.

During the additional measurement with the application of damping material B for the cavities of RHB 4 with thick ribs, no significant possibility of increasing the operating speed is found as compared with that of the body without the damping filling. The volume of the cavities is reduced by increasing the thickness of the ribs; such a reduced volume of added damping material has no clear benefit.

## 4. Re-Designed Absorber and Results

**Figure 8:**
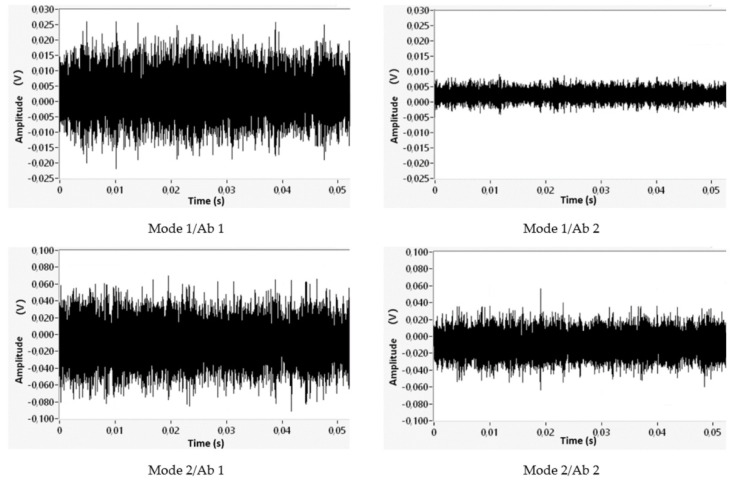
Time records of acoustic emission.

The results for the absorber components Ab 1 and Ab 2 in Figure 3 are presented using acoustic emission results, which were evaluated in the range from 50 to 400 kHz. We compared the acoustic emission at a steady excitation rotational speed of 135,000 min^−1^, which is the area of resonance for a rotor (Figure 8).

Even with the re-design of the absorber Ab 2, it is a matter of reducing the amplitudes of the dynamic response. In the time record of the acoustic emission between the native absorber Ab 1 and absorber with polymer concrete filling Ab 2, the range of amplitude values for excitation modes 1 (up to 5 g) and 2 (and above 10 g) (Figure 8) is reduced by 66% and 33%, respectively.

**Figure 9:**
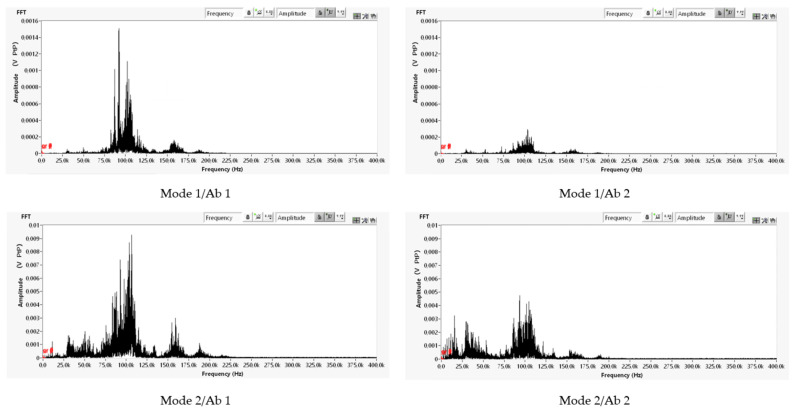
FFT analysis of acoustic emission.

In the fast Fourier transformation (FFT) spectrum (Figure 9) of acoustic emission, the dominant signal component is in the 100 kHz area. The maximum peak in the FFT spectrum is reduced by 85% and 50% for excitation modes 1 and 2, respectively, which is a significant appropriate change.

The heterogeneities are the main source of dissipation of vibration energy in microscale [8]. It is assumed that the heterogeneity of the polymer concrete by the presence of reinforcing particles contributes to the mentioned significant reductions in the acoustic emission values as compared with the application of the homogeneous damping materials A and B to the rotor housing body.

## 5. Summary and Conclusions

An experimental study was performed to reduce the amplitudes of high-frequency vibrations. The experiments included a bump test and determination of logarithmic decrement, measurement of vibration acceleration, wavelet transform, and measurement of acoustic emission. By creating a macroscopic hybrid structure of two machine tool joint components made of aluminium alloy and steel, and filling their cavities with polymers in the form of epoxy and silane resin and particulate composite (polymer concrete), we achieved a dynamic response, which was characterized by reduced amplitude values, reduced amplitude range, as well as changed natural frequency and damping parameters, resulting in a stabilisation of response and the possibility of increasing speed in the area of safe operation.

The use of a polymer filling represents a fast operative solution for improving the dynamic properties of the components of mechanical systems without the need for lengthy and expensive production of new components, for example, a casting. For the rotor housing body, there are significant benefits from the application of epoxy and silane damping materials (RHBs 2 and 3) for excitation up to 5 g, i.e., for the area of safe operation, namely:

- A reduction in the dynamic response for the area of safe operation, i.e., a decrease in the amplitudes of vibration acceleration by approximately 50% when using damping material;
- Response stabilisation, i.e., reductions in the range of amplitude values for the whole range of tested excitation frequencies.

By applying polymer concrete as a particulate composite in an absorber and for both operating modes (up to 5 g and above 10 g), for the acoustic emission, there is a reduction in the amplitude range by 66% and 33%, respectively, in the time domain, and a reduction in the peak of the FFT spectrum by 85% and by 50%, respectively, in the FFT analysis.

Another benefit of the damping material B is an increase in the first natural frequency, for the rotor housing body, by up to 90%. Furthermore, the logarithmic decrement values increased by 15% and 62%, respectively, when filling with damping materials A (RHB 2) and B (RHB 3) as compared with the native design (RHB 1). Considering the first natural frequency and the logarithmic decrement values, the silane damping material appears to be more advantageous, even though, in its case, it is a larger added volume and weight. Fillings with polymeric damping materials are clearly suitable for a spinning unit, which produce a reduction in vibration amplitudes, and a stable dynamic signal response.

Increasing the stiffness (thick ribs), which is a longer approach, shifted the operating speed by 40% and 10% for the warning and danger limits, respectively, when excited by large vibration amplitudes as compared with the native design of the rotor housing body with thin ribs. The application of the damping material for the body with thick ribs was not significant in terms of the possibility of increasing the operating speed. Due to the reduced volume of the cavities caused by the thicker ribs, its contribution was mainly in the stabilisation of the dynamic response.

For both components of the production machine joint filled with polymers and polymer concrete, such a re-design represented an improvement of dynamic properties as well as high-speed performance. In the next step, these re-designs will be implemented in production lines for long-term testing.

## Figures and Tables

**Table 1:** Materials and design properties.

	Materials	Mass *m* (gm)		Thickness of Ribs (mm)
		Separately	Together	
RHB 1	Aluminium alloy	579		2
RHB 2	Aluminium alloy	579 69	648	2
	Damping material A: epoxy resin in front cavities			
RHB 3	Aluminium alloy	579 191	770	2
	Damping material B: silane resin in front and back cavities			
RHB 4	Aluminium alloy	770		4
Ab 1	Steel	87		-
	Rubber: encased in bushing			
Ab 2	Steel	87 63	150	-
	Rubber: encased in bushing			
	Damping material C: polymer concrete in volume between bushings			

**Table 2:** Logarithmic decrement values.

	RHB 1	RHB 2	RHB 3	RHB 4
Logarithmic decrement	0.081	0.093	0.131	0.083

## Data Availability

Not applicable.
